# Investigations on 2Cr13 Stainless Valves after Dry-Type Laser Degumming

**DOI:** 10.3390/mi13050666

**Published:** 2022-04-24

**Authors:** Guang Li, Kai Li, Lu Zhang, Chen Liang, Chen Wang, Benhai Li, Junlong Wang, Xiaohua Wang, Mingwei Lei, Zhipeng Wei

**Affiliations:** 1 State Key Laboratory of High Power Semiconductor Laser, School of Physics, Changchun University of Science and Technology, 7089 Wei-Xing Road, Changchun 130022, China; liguang_21@163.com; 2 Beijing Institute of Aerospace Control Devices, Beijing 100094, China; jlulikai@163.com (K.L.); zhanglu0311@126.com (L.Z.); 18126203@bjtu.edu.cn (C.L.); wangchen960508@163.com (C.W.); libenhai@tom.com (B.L.); optics_wjl@163.com (J.W.)

**Keywords:** dry-type laser degumming, metal valve, metallographic structure, property variation

## Abstract

The disabled glue on valve surfaces is known to reduce aircraft durability and performance. In this paper, glue contaminants were removed from 2Cr13 stainless valves by dry-type laser processing with a cold air gun and compared with the chemical soaking method. The laser-processed surface was examined by white-light interferometer, scanning electron microscopy, energy dispersive spectroscopy, X-ray diffractometer, hardness tester, and metallographic microscopy. The substrate surface became a little smoother but also had deeper dips due to laser thermal melting. After laser degumming, the new constituent was found in the laser-irradiated region and analyzed as FeCr_0.29_Ni_0.16_C_0.06_, since the ratio of chemical compositions changed. Based on our simulation and experiments, the temperature of the workpiece was effectively controlled by the cold air gun, and its physical properties, including hardness and metallographic structure, were hardly changed. It was shown that laser degumming provides an alternative method for metal valve cleaning.

## 1. Introduction

As 2Cr13 stainless steel has the characteristics of high hardness, oxidation resistance, and corrosion resistance, it is often used to make transmission parts, valves, molds, and other structural parts [1,2]. 2Cr13 stainless valves are the main accessories of aircraft fuel systems, the surfaces of which are often covered in glue. As the basic components of glue are rubber, compounding agent and release agent, the glue easily ages over time. Therefore, 2Cr13 stainless valves must be degummed periodically as part of the maintenance process to guarantee the quality of the product. Traditional methods for degumming valves include burning or soaking with chemical reagent. In the first method, burning with fuel oil, it is not possible to accurately control the temperature, and the hardness of the workpiece can be significantly impacted. Meanwhile, the second method requires multiple soakings to soften the glue, and the contaminants need to be manually removed by knives after soaking, which can easily damage the substrate. Both methods, therefore, have problems, including the risk of chemical pollution, and are time-consuming and complex. Furthermore, these methods can lead to texture damage, deformation, and hardness reduction, which can increase the risks of poor sealing and even safety accidents.

Laser surface machining, as a pollution-free and high-efficiency technique, has been applied to various materials including plastics, metals, and their composites, and the results have been presented in various publications [3,4,5,6,7,8,9,10]. Rapid development of lasers has made it possible to perform surface modifications, such as coating removal and adhesive joints [11,12,13,14,15]. A continuous high-power semiconductor laser (HPDL) was used to remove rubber coating from a steel surface and the laser removal efficiency was as high as 250 mm^3^/s [16]. The tire mold was degummed by a 1064 nm Nd: YAG pulsed laser [17], as the cleaning threshold of rubber (of 22.0 J/cm^2^) was a little higher than the metal threshold (of 18.6 J/cm^2^) [18]. Lu et al. studied excimer laser cleaning of organic dirt on the IC die surface and found that the substrate damage threshold was ten times higher than the cleaning threshold of organic matter [19,20,21]. A CO_2_ laser with an average laser power of 14 W and laser scanning velocity of 880 mm/s was used to remove glue without damage to substrate of the sample [22]. Thus, laser degumming is promising in aerospace products over conventional processing methods, without causing thermal damage.

These studies mainly focused on how to efficiently remove glue below the damage threshold of metal; however, it is necessary to intensively study the influence of laser degumming on the substrate performance. As outlined in our previous essay [23], thermal effects from laser accumulation still exist, which can lead to changes in metallographic structures, matrix hardness, and even the destruction of the workpiece. The wet-type laser degumming method was difficult to achieve in engineering applications, as it generated waste liquid and caused secondary pollution [23]. In this paper, we conducted dry-type laser degumming experiments on 2Cr13 stainless valves using a cold air gun to achieve local cooling by heat convection with strong air flow. Results on surface morphology, roughness, material composition, metallographic structure, and matrix hardness of the metal substrate after degumming were investigated. Through the analysis of thermal simulation and experimental results, the feasibility of dry-type laser degumming technology applied to the surface of 2Cr13 stainless valves was demonstrated.

## 2. Materials and Methods

A. Sample preparation

**Figure 1:**
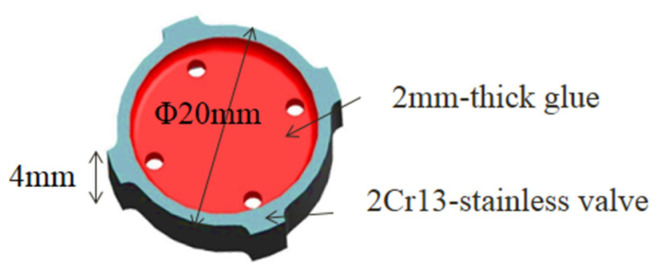
Schematic diagram of 2Cr13 stainless valve.

Figure 1 shows the schematic diagram of the valve sample with dimensions of Φ20 mm × 4 mm. The valve was made of 2Cr13 stainless steel with 2-mm-thickness glue covering its surface. The substrate was a composition of Fe and Cr, with smaller percentages of Mn, Si, Ni, C, and so on.

B. Experimental method

**Figure 2:**
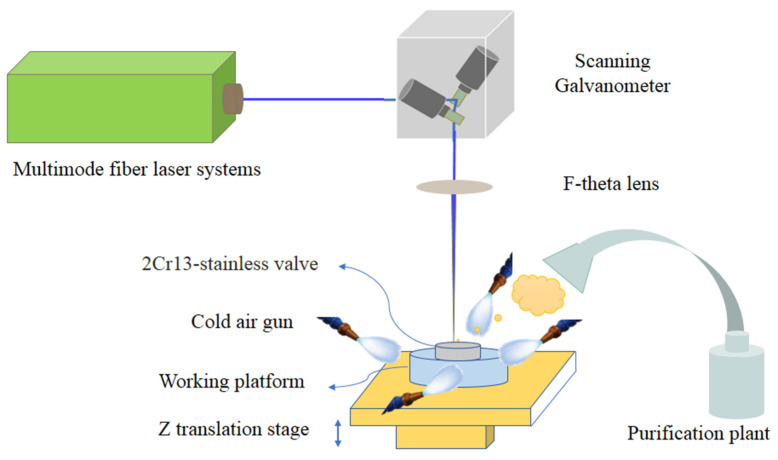
Schematic diagram of experimental setup for laser degumming.

A schematic diagram of experimental setup for laser degumming is presented in Figure 2. The experiments were performed using multimode fiber laser systems (IPG Company, New York, NY, USA) emitting high-order Gaussian laser beam with beam quality of M^2^ = 10, laser pulse duration of 100 ns, laser wavelength of 1064 nm, and a maximum average laser power of 200 W. Reflected by the scanning Galvanometer (Scanlab Company, Puchheim, Germany), the laser beam passed through an F-theta lens with a focal length of 210 mm. The laser beam was focused to be a spot with a radius of 300 μm (at 1/e^2^ of the maximum laser intensity) and a focusing depth of 10 mm. The distance between the laser spot and samples was controlled using a Z motorized translation stage assembly associated with EZCAD software. Furthermore, we utilized a cold air gun to produce strong air flow at the position of laser processing and achieved local cooling due to the heat convection. A purification plant was used to collect smoke and dust during the degumming process. As summarized in Table 1, the process parameters were determined after preliminary experiments, which were optimized for processing with higher degumming efficiency and better visual appearance. Considering energy efficiency, the laser power was set to the maximum of 200 W. Under high-intensity laser impact force, the glue was extruded out from the substrate surface. As the glue was too thick to clean in a single scan, laser scanning was applied 20 times to remove contaminants from samples with a laser scanning velocity of 2000 mm/s. As a result, the time for the glue removal process by means of laser treatment was about 1 min, which was one order of magnitude shorter than the commonly used method. For comparison, the degumming results via chemical soaking are also attached and labeled as sample NO.1, and sample NO.2 was degummed via laser treating.

C. Sample characterization

After degumming, the surface of the valve was firstly rinsed with alcohol to get rid of any impurities, and then the surface morphology, roughness, and material composition were measured and analyzed. An optical microscope (Olympus Company, BX51, Tokyo, Japan) was used to measure the sample topography. The surface roughness of the 2Cr13 stainless valve was measured by white light interferometer (ZYGO Company, Nexview9000, CA, USA). A scanning electron microscope (SEM, LEO Company, Leo-1450, Berlin, Germany) equipped with energy spectrometer (Kevex Company, SuperDry, New York, NY, USA) was utilized to measure the surface morphology of the substrate, and the capture of the energy spectrum to analyze its chemical composition was achieved by means of energy dispersive X-ray spectroscopy (EDX). An X-ray diffractometer (XRD, Rigaku Company, D/Max-RB, Tokyo, Japan) was used to identify the composition of the sample. The X-rays were produced from copper with monochromatic radiation of 0.154 nm, collimated and directed towards the sample. As the sample and the detector were rotated with a scanning speed of 2°/min, the intensity of the X-rays reflected from the top surface of the sample was recorded. Finally, in order to conduct metallographic observation and hardness test, the sample was cut into a small specimen with a size of 10 mm × 10 mm × 2 mm, inlaid in polymer resin, mechanically ground, subjected to polishing with emery paste, and electrolytic eroding with 10% oxalic acid solution was implemented to clearly identify the microstructure. Although the polishing procedure did change the micron-level thickness of the material, it did not alter the sample microstructure or hardness. The hardness of the substrate was measured with a micro-Vickers hardness tester (Everyone Company, Em-1500L, Shanghai, China), and the metallographic structures in the top and cross surface were measured with a metallographic microscope (Olympus Company, BX53M, Tokyo, Japan).

## 3. Results and Discussion

A. Analysis of surface morphology of 2Cr13 stainless valve after degumming

**Figure 3:**
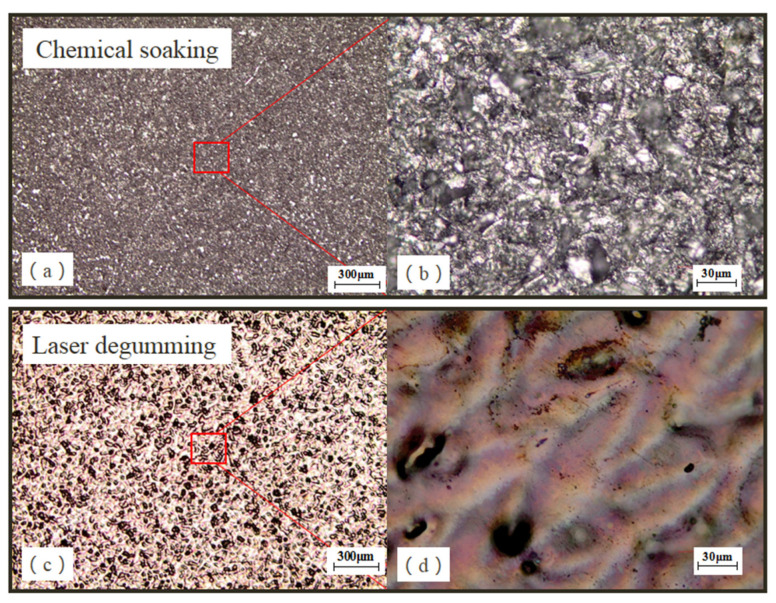
Sample topography done by chemical soaking (**a**,**b**) and laser degumming (**c**,**d**). Herein, (**a**,**c**) and (**b**,**d**) was detected by 50× and 500× optical microscopy, respectively.

The morphology of samples after degumming was captured by optical microscope and is shown in Figure 3. As chemical reagents only softened the glue and had no impact on the metal, the color of sample NO.1’s surface, processed by chemical soaking, was as grey as the original substrate, and its surface seemed a bit frosted due to the sandblasting process of the valve. Quite different from the former, sample NO.2, processed by laser degumming, turned to dark yellow and a large number of pits occurred on its top surface, which implied that the melting of the substrate took place during laser processing. Although the multimode laser cleaning technique was described to be non-destructive to metal materials because of the large spot size, the thermal accumulation from a long-lasting laser irradiation could affect its surface.

**Figure 4:**
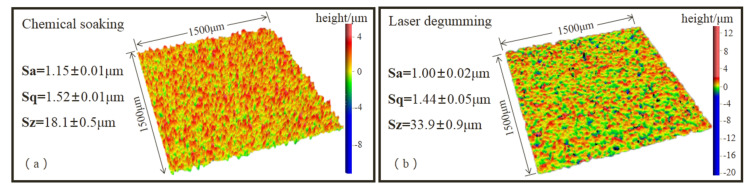
Sample surface roughness processing by chemical soaking (**a**) and laser degumming (**b**).

To get insight into the sample topography variation, the results measured by white light interferometer are presented in Figure 4. The sample surface after laser processing was a little smoother than after chemical soaking, as the values of Sa (the average of the height difference) and Sq (the root mean square of the height difference) were lower. On the other hand, the Sz value (the sum of the maximum peak height and valley depth) of the laser-processed sample was about twice as large as that of the chemical-processed sample, which was consistent with uneven pits seen in Figure 3d. In thermal ablation with long laser pulses [24], the absorbed laser energy heated the metal surface to the melting point, which produced a localized melt pool and made the attacked surface smooth. The center of the melt pool was rendered as a deeper pit, where some of the melted material would be squeezed out by laser impact force [25,26].

**Figure 5:**
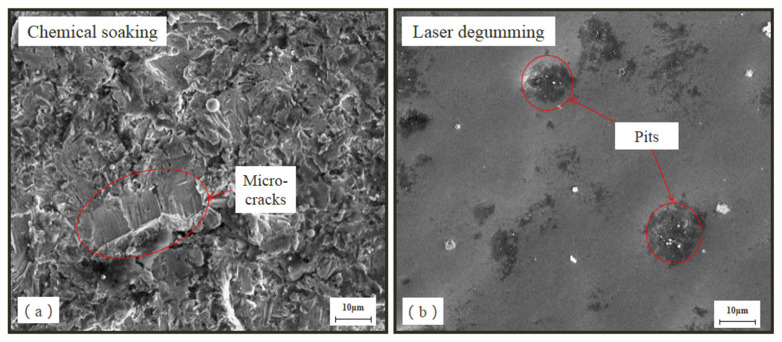
SEM micromorphology of the samples by chemical soaking (**a**) and laser degumming (**b**).

Figure 5 shows the microscopic topography of the substrate surface observed with 2000×-magnified scanning electron microscope. Due to manual scraping after soaking with chemical reagents, there were some micro-cracks on its surface, but the surface of sample NO.2 was smooth and without obvious mechanical damage, which was consistent with the results of surface roughness. Due to greater Sz roughness by laser processing, more pits, which were also deeper, appeared on the sample surface, which could increase the bonding area for the glue and improve the adhesivity on the re-gumming process.

B. Analysis of elemental components of 2Cr13 stainless valve after degumming

**Figure 6:**
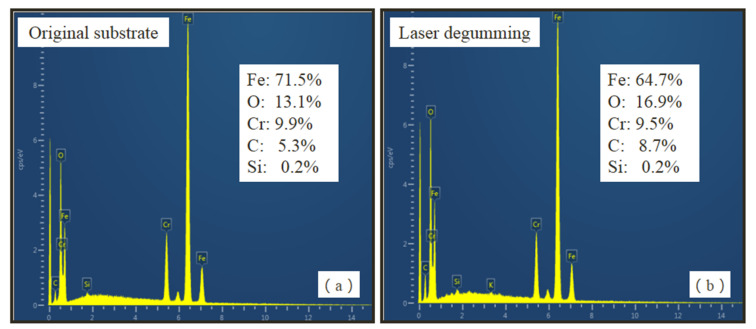
Energy spectrum of samples (**a**) before and (**b**) after laser degumming.

The main compositions of degummed samples were measured and are presented in Figure 6. The main chemical compositions of the original substrate were Fe (wt. 71.5%), O (wt. 13.1%), Cr (wt. 9.9%), and C (wt. 5.3%), where O content was from the oxidation of metal [27]. After laser processing, obvious changes in chemical composition of Fe and C were observed. Fe content decreased from wt. 71.5% to wt. 64.7%, whereas C content nearly doubled to wt. 8.7%. This implies that chemical reactions through carbonization took place during laser degumming, which brought new compounds into the sample.

**Figure 7:**
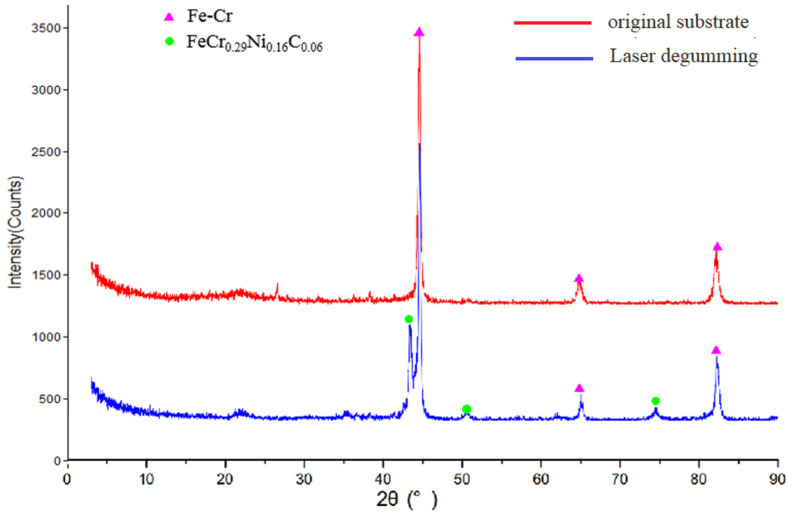
Composition test of samples before (red line) and after (blue line) laser degumming.

To discover the new compounds after laser degumming, we performed further measurement of the composition spectra by X-ray diffractometer, and the results are presented in Figure 7. According to Jade XRD database, these diffraction lines centered at 44.6°, 65°, and 82.3° were related to the surface phase of Fe-Cr, and the peaks at 43.5°, 50.6°, and 74.4° were assigned to FeCr_0.29_Ni_0.16_C_0.06_ [28]. After laser degumming, the main component of the laser-treated sample was still Fe-Cr and the same as the original substrate, but a small amount of FeCr_0.29_Ni_0.16_C_0.06_ was found on the sample surface. XRD measurements at different analyzed areas on the same sample were conducted and we confirmed that a small amount of FeCr_0.29_Ni_0.16_C_0.06_ was formed after laser degumming. The new layer of FeCr_0.29_Ni_0.16_C_0.06_ resulted from heat accumulation during laser processing. Following the high temperature of laser irradiation over a number of seconds, the chemical composition and the ratio of the metal surface changed during melting followed by solidification.

C. Analysis of physical properties of 2Cr13 stainless valve after degumming

**Figure 8:**
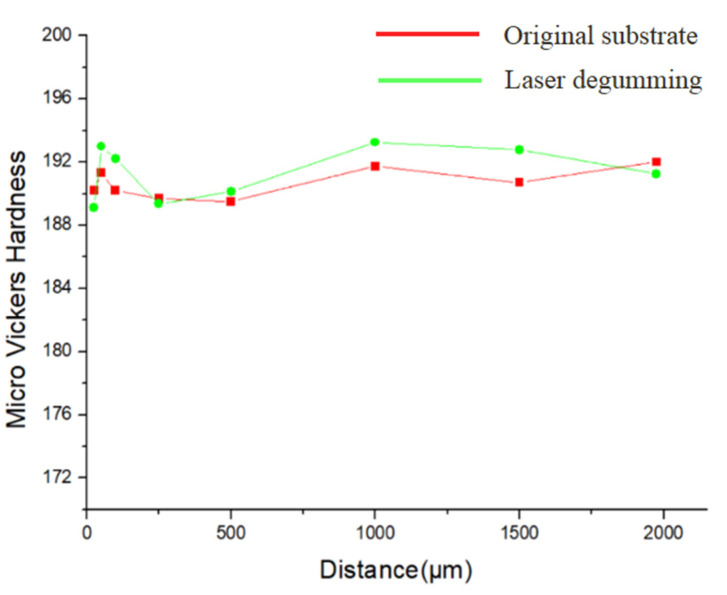
Micro-Vickers hardness as a function of the distance from the top surface.

In order to study the influence of new compound of FeCr_0.29_Ni_0.16_C_0.06_ on the physical properties of the workpiece, we measured the metal hardness as a function of the distance from the laser irradiated surface. The micro-Vickers hardness curves of the original substrate and sample after laser degumming are shown in Figure 8. Fortunately, the hardness values of the workpiece were almost unchanged from the top surface to the bottom of the laser-treated sample. Thus, no hardening layer was formed during laser processing [29], and the difference of metal hardness before and after laser degumming was within 1%. Since the sample hardness was basically the same as the original substrate, the influence of laser degumming on sample property was minimal, which guaranteed the quality of the workpiece.

**Figure 9:**
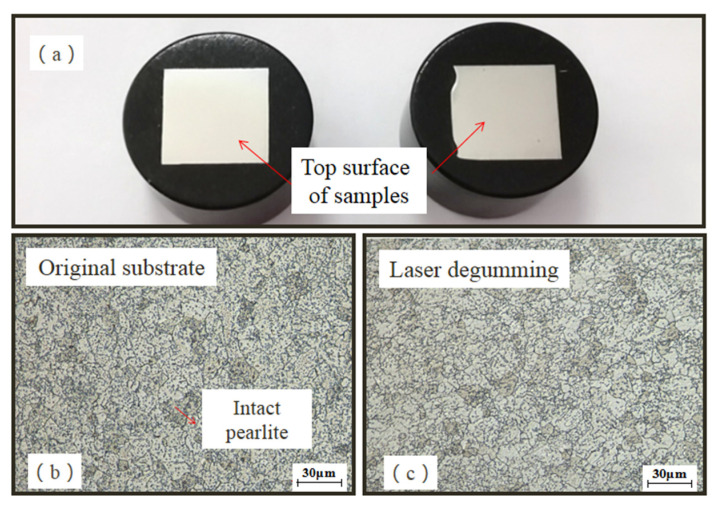
Sample preparation for metallographic examination (**a**) and micro-structures on the top surface of original substrate (**b**) and laser-degumming sample (**c**).

**Figure 10:**
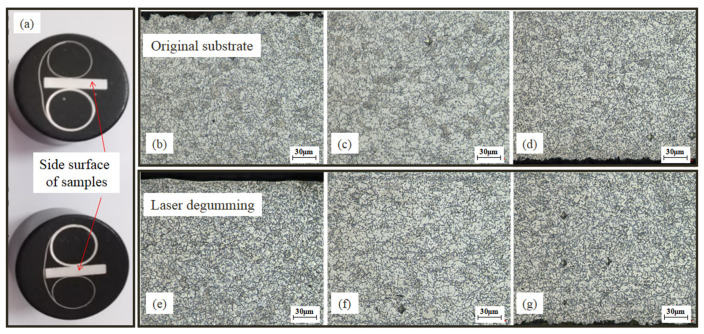
Sample preparation for metallographic examination (**a**) and micro-structures on the side surface of original substrate (**b**–**d**) and laser-degumming sample (**e**–**g**) at the different distances of 250 μm, 1000 μm, and 1750 μm away from the top surface, respectively.

To further study the new products on processed samples, metallographic structures of 2Cr13 stainless valve were measured. Before measurement, the sample was polished, which would get rid of micron-level thickness of material. As shown in Figure 9, the microstructures on the top surface of both the original substrate and the treated sample were the same as pelitic pearlites. Furthermore, Figure 10 shows the microstructures on the side surface in the different distances of 250 μm, 1000 μm, and 1750 μm away from the top surface. All were in uniform metallographic structures, and no martensitic tissue or transition layer was observed. Based on the results of metallographic examination, it was implied that the new layer of FeCr_0.29_Ni_0.16_C_0.06_ after laser degumming was as small as microns with a limited ability to change the property of the 2Cr13 stainless valve. Furthermore, this layer could easily be removed after sandblasting and would not interfere the re-gumming process.

D. Analysis of temperature field of 2Cr13 stainless valve during laser processing

**Figure 11:**
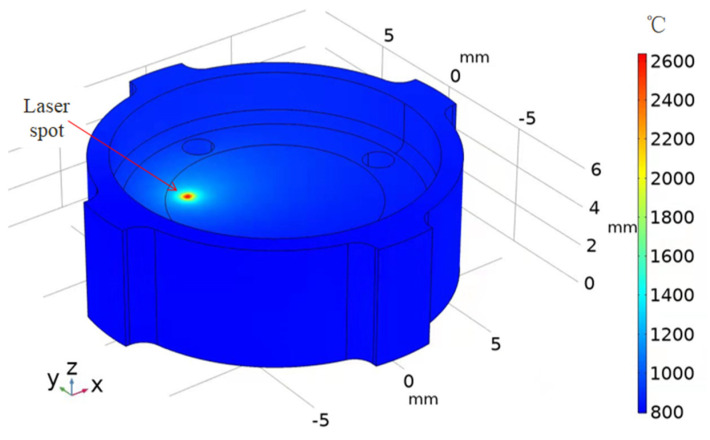
Temperature distribution of the workpiece at the end of laser processing.

To explain the microscopic topography variations after laser treatment, the temperature field of the 2Cr13 stainless valve during laser processing was simulated. We established a finite element model and analyzed the temperature field of the substrate. Considering the complexity of the cleaning process, to obtain a reliable temperature field simulation, we made the following simplifying assumptions:

(1) The substrate of the 2Cr13 stainless valve was isotropous and uniform.
(2) Laser heating of metal targets by long laser pulses was described as a nonlinear transient thermal model [30].
(3) The intensity of laser beam was described as follows:

(1) $$Q=\frac{2P}{\pi r_{0}^{2}}\cdot \exp\left(-2\cdot \frac{{\left(x-x_{0}\right)}^{2}+{\left(y-y_{0}\right)}^{2}}{r_{0}^{2}}\right)\cdot \left(1-R_{c}\right)$$

where P is the laser power; $r_{0}$ is the radius of laser spot; $R_{c}$ is the reflectivity of 2Cr13 stainless steel; and $x_{0}$ and $y_{0}\;$are, respectively, the coordinate values of the laser spot in the x and y directions. As the trajectory of the laser spot was designed as a constant-velocity spiral motion, we obtained $x_{0}=vt\cos\omega t$ and $y_{0}=vt\sin\omega t\;$; herein, v and ω are the scanning speed and angular velocity of the laser spot, respectively. As convective heat dissipation took place between the sample boundary and the air flow, we had heat transfer energy as $q_{0}=h\cdot \left(T_{0}-T\right)$; herein, h is the heat transfer coefficient and $T_{0}$ is the initial temperature of the workpiece. Relevant parameters used in the simulation are summarized in Table 2. The simulation results were calculated using COMSOL Multiphysics software, and the temperature distribution of the workpiece at the end of laser processing is shown in Figure 11 with the heat transfer coefficient of 20 W/(m^2^ K) in the atmospheric environment condition [31]. The temperature of the workpiece was highest as at the laser spot, i.e., ~2600 °C, which was higher than the melting temperature of stainless steel, i.e., ~1500 °C. As a result, the melted phenomenon occurred on its top surface with the moving of laser spot, which agreed well with our observed results. Because of good heat conduction of 2Cr13 stainless steel, the temperature sharply decreased with the distance away from the laser spot. Thus, the surface temperature at different positions was close except at the laser spot. After 60 s of laser irradiation, the workpiece would be heated overall. Since the surface temperature of the workpiece was highest on the surface and decreased gradually with increasing depth, the average temperature on the top surface needed to be considered.

**Figure 12:**
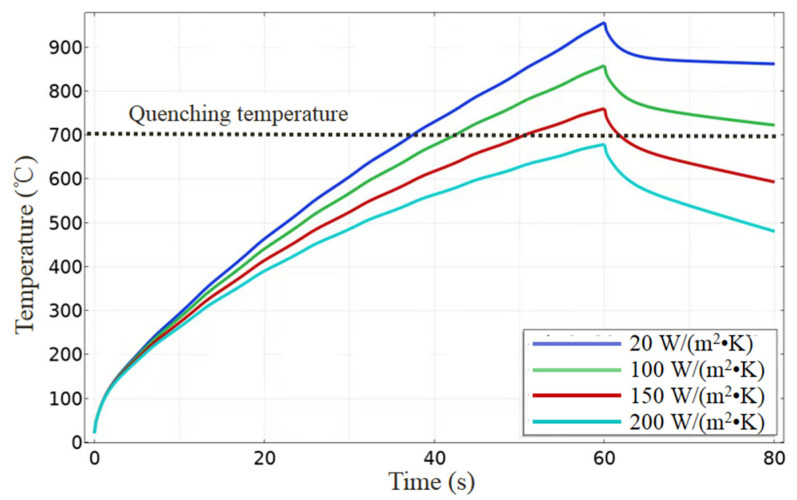
Variations of surface temperature with the time from laser processing. Herein, the heat transfer coefficients were 20 W/(m^2^ K) (blue line) for the atmospheric environment condition and 100 W/(m^2^ K) (green line), 150 W/(m^2^ K) (red line), and 200 W/(m^2^ K) (cyan line) for the strong air flow condition.

In order to estimate the heat accumulation from the laser beam, the average temperature on the top surface was used to characterize the surface temperature. We present the variations of the surface temperature with the time from the laser processing in Figure 12. The workpiece firstly heated under thermal ablation with long laser pulses, and then the increase became slow due to heat conduction into the bulk, and finally the temperature got lower until the laser was turned off. Under the atmospheric environment conditions, the workpiece was heated high enough to reach its quenching temperature of 700 °C [32], and the sample properties changed greatly, which was reported in the previous essay [18]. Under strong air flow, the heat transfer coefficient could be increased dozens of times, and the theoretical results for 5–10 times are also shown in Figure 12. Because of strong air convection between the workpiece and air flow, the maximum temperature of the workpiece was reduced obviously; meanwhile, the workpiece was cooled down in a higher velocity. With the help of a cold air gun, the surface temperature was effectively controlled and ensured to be less than the quenching temperature of the substrate. As a result, the sample properties after laser processing hardly changed, which is consistent with our experiment results.

## 4. Conclusions

Comparing with the traditional chemical reagent soaking, we conducted dry-type laser degumming experiments using a cold air gun. Experimental and theoretical results on variations of surface morphology, elemental components, physical properties, and temperature field of 2Cr13 stainless valves were presented. Because of heat accumulation from the laser beam, the sample was heated significantly and the micron-level thickness of the FeCr_0.29_Ni_0.16_C_0.06_ layer was found on the laser-treated surface. According to our simulation and experiments, the surface temperature was effectively cooled down below its quenching temperature by cold air gun and the hardness variations of the workpiece was less than 1%. As a result, dry-type laser degumming is promising in achieving high-efficiency and good-quality removal of glue on the surface of metal press valves.

## Figures and Tables

**Table 1:** Experimental parameters.

Parameter	Values
Laser power (W)	200
Repetition frequency (kHz)	20
Scanning velocity (mm/s)	2000
Scanning times	20
Method	Optimization

**Table 2:** Parameters used to simulate temperature field during laser processing.

Parameters	P (W)	r_0_ (μm)	R_c_ (%)	v (mm/s)	*ω* (rad/s)	T_0_ (°C)
Values	200	300	70	2000	10,000	20
